# Biophysical stimulation of bone and cartilage: state of the art and future perspectives

**DOI:** 10.1007/s00264-018-4274-3

**Published:** 2019-01-15

**Authors:** Leo Massari, Franco Benazzo, Francesco Falez, Dario Perugia, Luca Pietrogrande, Stefania Setti, Raffaella Osti, Enrico Vaienti, Carlo Ruosi, Ruggero Cadossi

**Affiliations:** 1 University of Ferrara, Via Vigne 4, 44121 Ferrara, Italy; 2 IRCCS Foundation “San Matteo” Hospital, University of Pavia, 27100 Pavia, Italy; 3 Santo Spirito in Sassia Hospital, 00193 Rome, Italy; 4 La Sapienza University, 00185 Rome, Italy; 5 San Paolo Hospital, University of Milan, 20142 Milan, Italy; 6 IGEA Clincal Biophysics, 41012 Carpi, MO Italy; 7 Osti Clinic, 44121 Ferrara, Italy; 8 University of Parma, 43100 Parma, Italy; 9 Federico II University Naples, 80100 Naples, Italy

**Keywords:** Biophysical stimulation, Bone tissue, Cartilage, PEMF, CCEF, LIPUS

## Abstract

**Introduction:**

Biophysical stimulation is a non-invasive therapy used in orthopaedic practice to increase and enhance reparative and anabolic activities of tissue.

**Methods:**

A sistematic web-based search for papers was conducted using the following titles: (1) pulsed electromagnetic field (PEMF), capacitively coupled electrical field (CCEF), low intensity pulsed ultrasound system (LIPUS) and biophysical stimulation; (2) bone cells, bone tissue, fracture, non-union, prosthesis and vertebral fracture; and (3) chondrocyte, synoviocytes, joint chondroprotection, arthroscopy and knee arthroplasty.

**Results:**

Pre-clinical studies have shown that the site of interaction of biophysical stimuli is the cell membrane. Its effect on bone tissue is to increase proliferation, synthesis and release of growth factors. On articular cells, it creates a strong A_2A_ and A_3_ adenosine-agonist effect inducing an anti-inflammatory and chondroprotective result. In treated animals, it has been shown that the mineralisation rate of newly formed bone is almost doubled, the progression of the osteoarthritic cartilage degeneration is inhibited and quality of cartilage is preserved. Biophysical stimulation has been used in the clinical setting to promote the healing of fractures and non-unions. It has been successfully used on joint pathologies for its beneficial effect on improving function in early OA and after knee surgery to limit the inflammation of periarticular tissues.

**Discussion:**

The pooled result of the studies in this review revealed the efficacy of biophysical stimulation for bone healing and joint chondroprotection based on proven methodological quality.

**Conclusion:**

The orthopaedic community has played a central role in the development and understanding of the importance of the physical stimuli. Biophysical stimulation requires care and precision in use if it is to ensure the success expected of it by physicians and patients.

## Biophysical stimulation

Clinical biophysics forms the foundation of a “new pharmacology” which uses physical stimuli to treat various diseases in human beings. Biophysical stimulation techniques can be used in clinical medicine, either alone, to increase and promote the repair and anabolic activity in tissue, or in association with drug treatment, to strengthen its activity and lessen side effects. Clinical biophysics is an interdisciplinary science which:

- Uses methods and theories from the field of physics to study biological systems
- Studies how non-ionising physical stimuli interact with biological systems

The medical community is certainly familiar with the idea of modifying the behaviour of a certain cellular activity using a chemical agent or macromolecules, such as growth factors, genes or a part of these. The ability to modify the activity of a biological target using a physical agent, however, is a more recent and unfamiliar discovery.

The ability of biological systems to absorb energy initially led researchers to focus their attention on dose as the fundamental parameter on which “all” the effects depend. Research then moved to the biological effects dependent not only and not so much on the total energy introduced into the system, as on the other properties which describe the physical agent: frequency, amplitude and the form of the signal wave.

The complexity of the interaction between physical agents and biological systems has made the researchers’ work particularly difficult, and it is only today that we have acquired a degree of knowledge such that physics can significantly help the development of biology and lead to the opening of new horizons where the clinical use of physical means is concerned.

The methods for administrating physical energy to a biological system are known as biophysical stimulation and can be divided into electrical energy applied directly to the tissue using adhesive electrodes (capacitively coupled electrical field, CCEF), electromagnetic energy applied using coils (pulsed electromagnetic fields, PEMFs) and ultrasound energy applied directly to the tissue in the form of mechanical forces (low intensity pulsed ultrasound system, LIPUS).

**Fig. 1:**
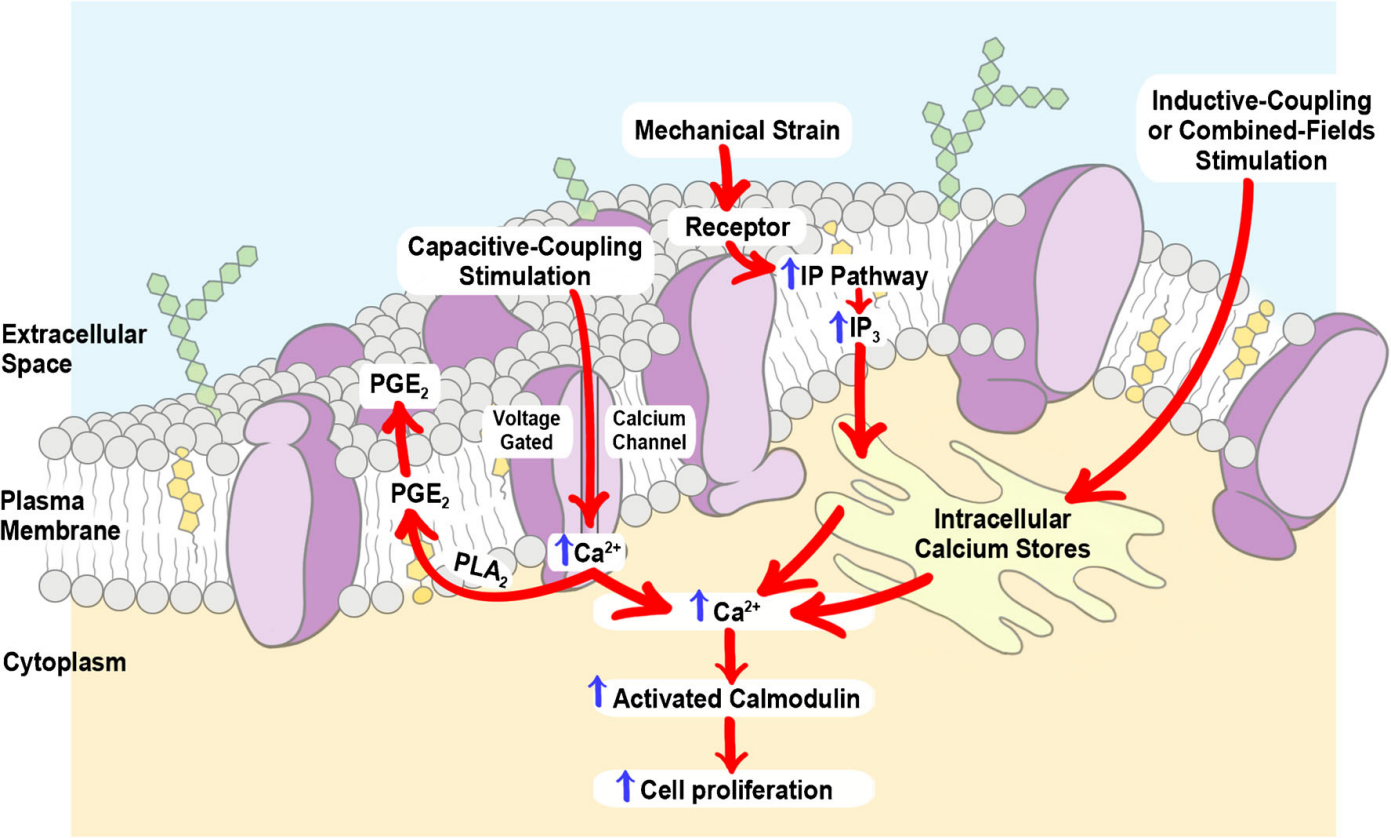
Schematic representation of the biophysical stimuli targets on the cell surface and corresponding metabolic pathways within the cell

Underlying the new pharmacology is the need to identify the effects of the physical agents in terms of how these modulate a particular cell function, which will then form the basis of its clinical application. The cell membrane has been identified as a target and site of interaction, through which the physical signal activates a cascade of intracellular events; the transduction pathways have been seen to differ depending on the type of energy used (Fig. 1). Each time a physical agent is able to modulate a cell activity, the effect observed will be function-specific, rather than cell- or tissue-specific. This allows all conditions which are positively influenced by the activation or modulation of this cell function to be treated with the same physical agent.

The key principles of biophysical stimulation are as follows:

1. The ability of the physical stimulus to act selectively on cell targets
2. Signal specificity, i.e. the effect depends on waveform, frequency, duration and energy
3. Identification of the dose-response effects
4. The signal should maintain the characteristics identified as being effective at the disease site.

Knowledge of the mechanism of action should provide the rational basis for clinical application, permitting the clinical studies and relative end-points to be designed in a coherent manner.

## Biophysical stimulation: in vitro studies

### Effects of biophysical stimulation on bone cells

**Fig. 2:**
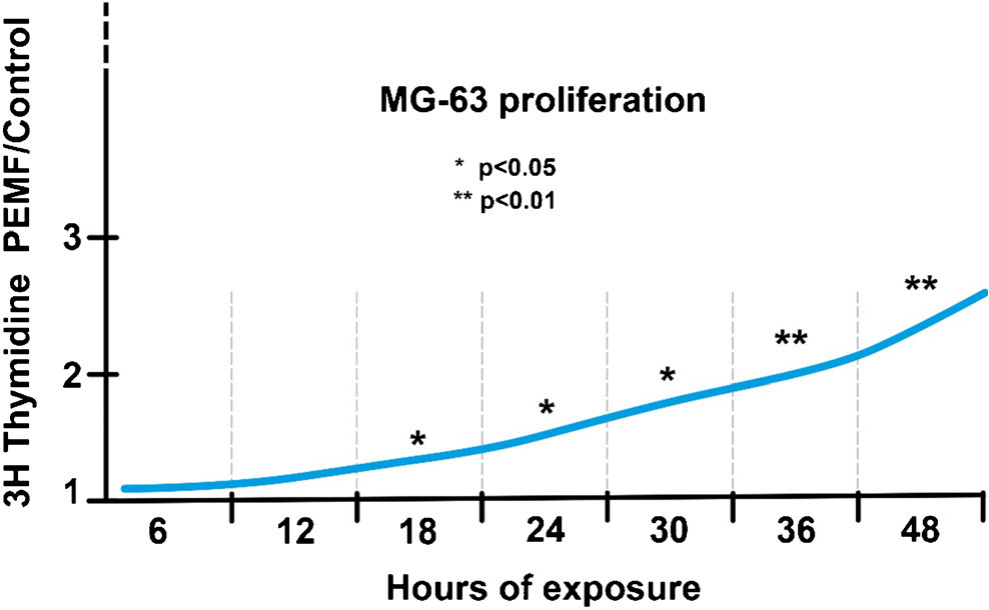
Effect of PEMF exposure length on human osteosarcoma cell lines and human osteoblast cell (MG63) proliferation (Sollazzo et al. Electricity and Magnetism in Biology and Medicine 1997)

Numerous studies have analysed the effects of biophysical stimulation on osteoblast proliferation and have highlighted a dose-response effect for exposure times, PEMF intensity, frequencies and signal waveforms [1, 2] (Fig. 2). More specifically, Brighton et al. [3] showed that a CCEF of 0.1–10 mV/cm stimulates the proliferation of rat calvarial bone cells, while lower intensities proved ineffective. In human osteosarcoma cell lines and osteoblast cells in vitro, De Mattei et al. [4] identified a relationship between PEMF exposure times and the increase in proliferation, as well as differences in exposure times between different cultures. Leung et al. [5] studied the effect of LIPUS on cell cultures of human periosteal cells, along with the effects in relation to time and dosage. Total number of live cells, cell proliferation, alkaline phosphatase activity, osteocalcin secretion and expression of vascular endothelial growth factors were evaluated. The authors demonstrated a clear dose-dependent effect, the greatest efficacy being recorded at 20 minutes exposure. Similar to that reported for the proliferative effects, in vitro studies have shown that in various cell models, biophysical stimulation induces (*i*) an increase in osteoblast differentiation, promoting the production of collagen and of the main matrix glycoproteins osteocalcin and osteopontin [6, 7]; (*ii*) stimulates the mineralisation process [8, 9]; and (*iii*) plays an inhibitory role in the process of osteoclast differentiation and exerts a protective action against osteolysis [10]. The increases induced by biophysical stimulation in the production of bone matrix are very similar to those induced by growth factors such as TGF-β1, BMPs and growth factor IGF-I, indicating that the effects induced by a biophysical stimulus are of significant medical importance [11, 12] (Table 1).

**Table 1:** Biophysical stimulation on the regulation of bone matrix and growth factors

Author	Physical method	In vitro models	Results
Jansen JH, BMC *Musculoskelet Disord*. 2010	PEMFs	hBMSCs	↑ TGF-β1 ↑ BMP-2mRNA ↑ Differentiation
Esposito M, *In Vivo*. 2012	PEMFs	hBMSCs	↑ Proliferation ↑ Differentiation
Ceccarelli G, *Biores Open Access*. 2013	PEMFs	hBMSCs	↑ Proliferation ↑ ECM deposition
Zhou J, *Bioelectromagnetics*. 2013	PEMFs	Rat calvarial osteoblasts	↑ Proliferation
Hartig M, *Eur Biophys J*. 2000	CCEF	Osteoblast from periosteum explants	↑ Proliferation ↑ Differentiation
Wang Z, *J Bone Joint Surg Am*. 2006	CCEF	Osteoblastic cells (MC3T3-E1)	↑ BMP-2,4,5,6,7 mRNA
Bisceglia B, *Bioelectromagnetics*. 2011	CCEF	Osteoblast-like cell lines (SAOS-2)	↑ Proliferation
Clark CC, *J Orthop Res*. 2014	CCEF	Human calvarial osteoblasts	↑ BMP-2,4 mRNA ↑ TGF-β1, β2, β3 mRNA ↑ FGF-2
Hauser J, *J Orthop Res*. 2009	LIPUS	Osteoblast-like cell lines (SAOS-2)	↑ Proliferation
Fassina L, *Bioinorg Chem Appl*. 2010	LIPUS	SAOS-2 human osteoblasts	↑ Proliferation ↑ ECM deposition
Xue H, *PLoS One*. 2013	LIPUS	Alveolar bone in vivo	↑ BMP-2 mRNA
Carina V, *J Appl Biomater Funct Mater*. 2017	LIPUS	Human mesenchymal stem cells	↑ Proliferation ↑ MgHA/coll hybrid composite scaffold ↑ VEGF gene expression

### Effects of biophysical stimulation on articular cells

Extensive in vitro data reported in literature shows the effect of PEMFs on articular cells (Table 2). In bovine chondrocytes and synoviocytes [13, 14], A_2A_ and A_3_ adenosine receptors, endogenous modulator of many biological processes such as inflammation, increased in number in the presence of PEMFs, reducing the release of PGE_2_, IL-6, IL-8 and COX-2, a result which suggests a reduction in the inflammatory state and in the degradation of cartilage associated with articular diseases. Human synoviocytes treated with PEMFs reveal a significant increase in A_2A_ and A_3_ adenosine receptors, as demonstrated by the mRNA, Western blotting analysis and saturation binding experiments involving ARs, as well as a significant increase in the release of IL-10, a known anti-inflammatory cytokine [15]. The A_2A_ and A_3_ receptors exert their anti-inflammatory action by inhibiting the NF-κB transcription factor pathway, which plays a central role in regulating the synthesis and activities of the inflammatory cytokines. Stimulation with PEMFs further inhibits the activation of NF-κB and is essential for regulating the synthesis and activation of the pro-inflammatory cytokines, including TNF-α and IL-1β, and also of other mediators involved in joint inflammation and bone diseases [16]. PEMFs have been shown to affect the increase of human articular chondrocyte proliferation, based on exposure time, intensity and frequency [17]. It should be stressed that the effect of PEMFs on proteoglycan synthesis in human cartilage explants is comparable in all senses to that induced by growth factor IGF-I, the principal cartilage anabolic factor [18, 19]. While the presence of IL-1β inhibits the synthesis of proteoglycans, exposure to PEMFs can curb the catabolic effect of the cytokine, increasing proteoglycan synthesis even under inflammatory conditions [20]. It is interesting to observe that these results regarding the anti-inflammatory role of PEMFs are also confirmed in the stem cell cultures [21].

**Table 2:** Pulsed electromagnetic field effect in articular cells

Culture	PEMF effects
Bovine chondrocytes and synovial fibroblasts	Increase of A_2A_ and A_3_ receptors
	Increase of cellular proliferation
	Inhibition PGE_2_ release
Bovine articular cartilage explants	Increase of proteoglycan synthesis
	Chondroprotective effect
Human synovial fibroblasts	Inhibition of PGE_2_ IL-6, IL-8, and TNF-α release
	Stimulation of IL-10 release
Human articular cartilage explants	Increase of proteoglycan synthesis
	Counteract the catabolic activity of IL-1b
	Increase of cartilage explant anabolic activities
Human T/C-28a2 chondrocytes and hFOB 1.19 osteoblasts	Increase of A_2A_ and A_3_ receptors
	Inhibition of PGE_2_ IL-6, IL-8, and VEGF release
	Increase of cellular proliferation
	Increase of osteoprotegerin (OPG) production
	Inhibition of NF-κB activation
	Reduction of cAMP levels

## Biophysical stimulation: in vivo studies

### Effects of biophysical stimulation on bone repair

**Fig. 3:**
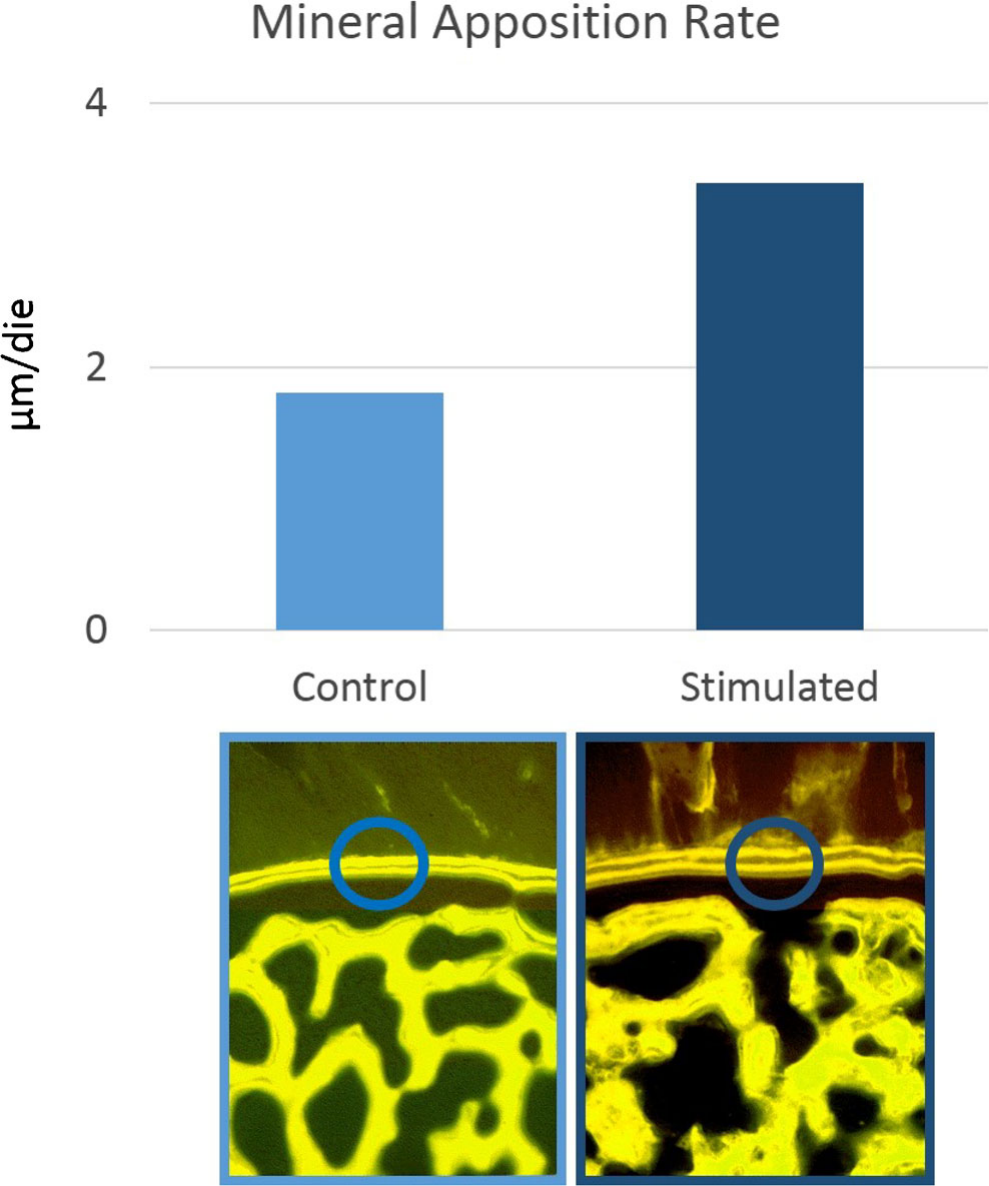
Effect of PEMF stimulation on mineral apposition rate in newly formed trabeculae measured by tetracycline labelling, in transcortical holes bored in the distal metaphysis and diaphysis of the third metacarpal bone in horses (Canè V et al. J Orthop Res 1993)

**Fig. 4:**
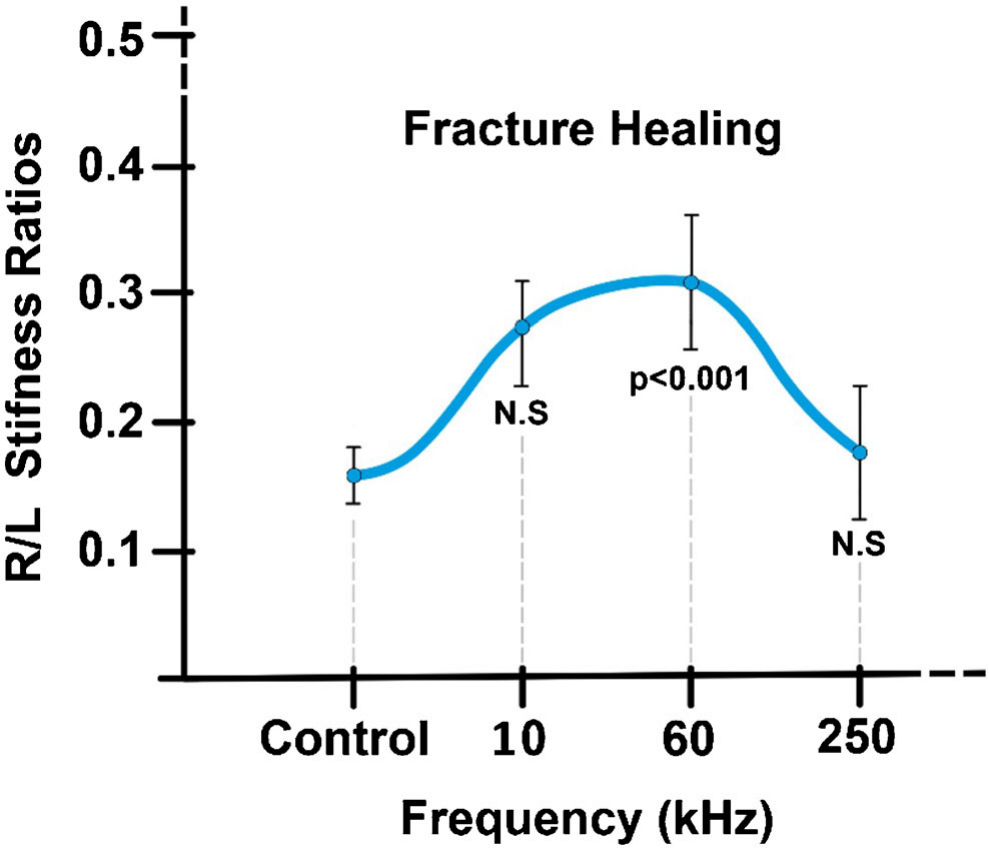
Effect of CCEF frequency on fibula osteotomy healing in rabbits (Brighton CT et al. J Orthop Res 1985)

There are numerous studies in literature, performed in animal models as early as the 1970s, which attest to the osteogenic effects of biophysical stimulation in bone repair. In a 1974 study on dogs, Bassett et al. [22] demonstrated the effect of PEMFs in stimulating the repair of a bilateral fibular osteotomy, indicating the need to carry out further studies to ascertain the physical characteristics of the signal most effective in obtaining the biological effect desired. De Haas et al. [23] also published a study whose objective was to evaluate the effect of PEMFs at different frequencies in an experimental rabbit osteotomy model, showing that the treatment does not cause pathological alterations to the tissues and that different signal characteristics could result in different outcomes. In transcortical holes bored in the distal metaphysis and diaphysis of the third metacarpal bone in horses, the histological results demonstrate that PEMFs increase the quantity of newly deposited bone inside the hole from 40 to 120% more compared to the controls and that the amount of newly deposited bone and mineral apposition rate inside the holes are significantly greater in the treated limbs compared to controls [24] (Fig. 3). In 2005, Midura et al. [25] exposed rat osteotomies to PEMF stimulation at two different frequencies and intensities (15 Hz, 2 mT vs 1.5 Hz, 0.02 mT) with similar results, showing that the higher frequency (15 Hz) treatment led to a doubling of both the apposition rate and the volume of the bone callus. The importance of the signal characteristics was also studied for the CCEF and LIPUS methods. In a 1985 study involving a rabbit fibular osteotomy model, Brighton et al. [26] showed that only the 60-KHz frequency, compared to those at 10 and 250 KHz, gave significantly better results than the controls (Fig. 4). In 1994, Rijal et al. [27] created a non-union experimental model, where a DEXA bone densitometry scan showed an increase in density of 18% (*p* < 0.05) in the CCEF group compared to the controls. In the 1980s, Duarte [28] used LIPUS at a frequency of 1.5 MHz with intensity of 30 mW/cm^2^ applied for 15 minutes/day in a rabbit fibular osteotomy model, obtaining an increase of 28% in ossification in the limbs treated. A series of in vivo studies followed in the 1990s, giving a better understanding of the ultrasound signal characteristics and treatment times able to accelerate the bone healing process in fractures. In bilateral rabbit fibular osteotomies, Pilla [29] demonstrates that the application of LIPUS (200 μs impulses at 1.5 MHz, at an intensity of 30 mW/cm^2^ for 20 min/day) accelerates bone healing by a factor of 1.7 compared to controls in terms of mechanical resistance. The LIPUS applied have characteristics that have already been approved by the FDA for use in clinical medicine for 20 minutes/day.

As clearly emerges from the studies illustrated, obtaining the desired biological effect depends on the type of signal used. For this reason, basic pre-clinical research is fundamental for identifying the physical characteristics of the stimulus able to interact with the biological system to be influenced and in defining its value for clinical practice.

### Effects of biophysical stimulation on articular cartilage

Studies based on in vitro and ex vivo results have been performed on large and small animal models to evaluate the effect of PEMFs in preventing osteoarthrosic degeneration and in the repair of tissue damage, as an adjunct to tissue engineering methods.

In Dunkin Hartley guinea pigs [30], treatment with PEMFs was demonstrated as being capable of halting the progression of osteoarthrosis, of limiting cartilage surface clefts and fibrillation, of preserving cartilage thickness and of preventing sclerosis of the subchondral bone. These results are coherent with those of other authors, who have demonstrated an increase in TGF-β1 synthesis and an inhibition of TNF-α synthesis (with a clear anabolic and trophic effect on the articular cartilage) in the animals treated using biophysical stimulation. Autologous osteochondral autografts were performed in adult sheep [31], resulting in a significantly better osteointegration of the graft and a lesser formation of cyst-like resorption areas in the PEMF group. The synovial liquid in the stimulated animals contained significantly lower levels of pro-inflammatory cytokines IL-1β and TNF-α and a higher concentration of TGF-β1 compared to the untreated animals. PEMFs have proved effective in rabbits with osteochondral lesions [32], significantly improving the quality of the regenerated tissue in the osteochondral defects in the presence of collagen scaffold and bone marrow concentrate.

The anti-inflammatory activity of PEMFs effectively prevented the degenerative effect of IL-1β, significantly improving cartilage regeneration compared to the non-stimulated lesions, thus explaining the anti-degenerative, reparative and anti-inflammatory effects of treatment with PEMFs in in vivo models also.

## Biophysical stimulation: clinical experiences on bone tissue

### Osteotomy

**Fig. 5:**
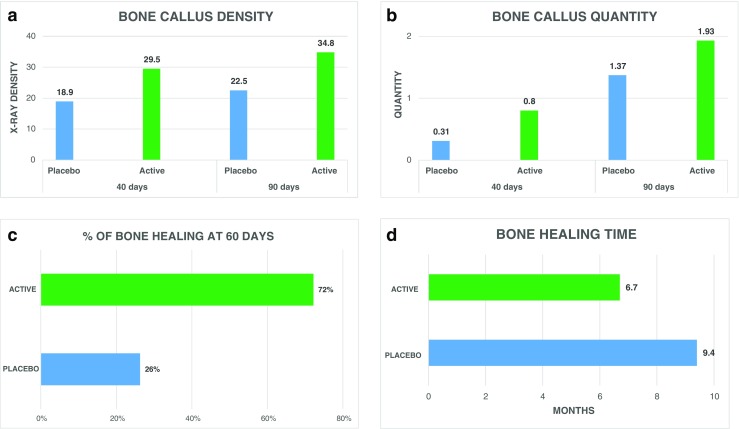
Effect of PEMF (**a**, **b**) on femoral osteotomies at 40 and 90 days from surgery (Borsalino G et al. Clin Orthop Relat Res 1988), (**c**) on tibia osteotomies (Mammi GI et al. Clin Orthop Relat Res 1993) and (**d**) in patients undergoing allograft reconstruction following tumour resection (Capanna R et al. Clin Orthop Relat Res 1994)

The first studies on the effect of biophysical stimulation recognised as having level of evidence I status were three in number and were conducted by the Italian orthopaedic community as early as the 1980s, on patients undergoing osteotomy of the lower limbs. The studies report a significant increase in the density of the trabecular bone callus in the active group compared to the placebo group [33], a higher consolidation success rate [34] and a shortening of consolidation time by three months [35] (Fig. 5).

### Fractures at risk of non-union

Biophysical stimulation has proved capable of accelerating the healing of “at-risk” fractures treated by plaster casting or internal or external fixation or complex fractures with serious damage to soft tissues and exposure of bone tissue [36, 37]. In patients with femur neck fractures, Faldini et al. [38] report a percentage of healing of 94% in the PEMF group compared to 69% in the placebo group, while Benazzo et al. [39], using the CCEF technique on athletes with stress fractures, observed a reduction in functional recovery times with a success rate of 88%. In patients with fractures immobilised using plaster casting, external fixator or intramedullary nails, various authors report a significant reduction of 38% in healing times using LIPUS [40–43].

### Non-unions

There is abundant clinical evidence in international literature supporting the efficacy of biophysical stimulation on non-union fractures, particularly with PEMFs; authors have reported success rates of around 70–80% [44, 45] (Table 3). Good results have also been obtained in the treatment of non-union fractures with CCEFs and with LIPUS, albeit on a lesser scale.

**Table 3:** Summary of main clinical studies using pulsed electromagnetic field therapy in non-unions

Author	Design of the study	Non-union, treatment	Groups	Number	Results: success rate and healing time
De Haas WG, *J Bone Joint Surg Br*. 1980	Case series	Tibial non-union, cast	Stimulated	17 patients	88.2% in 5.9 months
Bassett CA, *J Bone Joint Surg Am*. 1981	Case series	Tibial non-union	Stimulated	125 patients	87%
Simonis RB, *Injury* 1984	Case series	Non-union of long bone, external fixator	Stimulated	15 non-unions	87% in 4 months
Sedel L, *Rev. Chir Orthop Reparatrice Appar Mot*. 1981	Case series	Non-union, different treatment	Stimulated	37 patients	83%
Bassett CA, *JAMA*. 1982	Case series (cross-sectional international study)	Non-union and failed arthrodesis	Stimulated	1007 non-unions, 71 failed arthrodesis	85%
Sharrard WJ, *J Bone Joint Surg Br*. 1982	Case series	Non-union of tibia, femur, ulna, radius, humerus, capitellum, knee, ankle	Stimulated	53 non-unions	71.7% (86.7% tibia) in 6 months
Marcer M, *Clin Orthop Relat Res*. 1984	Case series	Non-union of tibia, femur, humerus, external fixator	Stimulated	147 patients	73%
Hinsenkamp M, *Reconstr Surg Traumatol*. 1985	Case series	Non union	Stimulated	308 patients	70%
Frykman GK, *J Hand Surg [Am]*. 1986	Case series	Non-united scaphoid fracture, cast	Stimulated	44 non-unions	79%
Traina GC, *Giornale Italiano di Ortopedia.* 1986	Case series	Non-union, cast, external fixator, other	Stimulated	248 patients	84% in 4.3 months
Garland DE, *Contemp Orthop*. 1991	Case series	Non-union, external and fixator	Stimulated	139 non-unions	80% (> 3 h/die) vs 35.7% (< 3 h/die) in 12 weeks
Gupta AK, *Indian J Orthop*. 2009	Case series	Tibial non-union, cast	Stimulated	45 fractures	85%, in 4 months
Assiotis A, *J Orthop Surg Res*. 2012	Case series	Tibial non-union, plates, nail, plaster of Paris	Stimulated	44 patients	77.3%
Punt BJ, *Eur J Orthop Surg Traumatol.* 2008	Prospective comparative study	Non-union of long bone, non-long bone cast, external fixator, other	Stimulated (long bone vs non-long bone)	93 patients	76 vs 79%
Cebrian JL, *International Orthopaedics*. 2010	Prospective comparative study	Tibial non-union, intramedullary nailing	Stimulated vs stimulated + surgery	22 vs 35 patients	91 vs 83%, in 3.3 vs 4.9 months
Poli G, *J Bioelectricity*. 1985	Randomised controlled double-blind study	Congenital non-union, endomedullary nail fixation	Stimulated vs surgery	6 vs 6 patients	Lengthening of the limb, stop imbalance between legs
Sharrard WJ, *J Bone Joint Surg Br*. 1990	Randomised controlled double-blind study	Tibial non-union, cast	Active vs placebo	20 vs 25 fractures	45 vs 12% at 12 weeks
Simonis RB [44], *Injury.* 2003	Randomised controlled double-blind study	Tibial non-union, osteotomy, and external fixator	Active vs placebo	18 vs 16 patients	89 vs 50%
Shi HF, *BMC Musculoskelet Disord*. 2013	Randomised controlled double-blind study	Non-union of long bone, nail, plate	Active vs placebo	31 vs 27 non-unions	77.4 vs 48.1% in 4.8 months
Traina GC [45], *J Bioelectricity*. 1991	Retrospective controlled	Non-union leg, femur, forearm, humerus, metatarsal, clavicle different treatment	Stimulated vs surgery	41 vs 26 patients	87.8 vs 69% in 5.7 vs 7.8 months
Vaquero DH, *Revista de ortopedia y Traumatologia.* 2000	Retrospective cohort	Non-union tibia, femur, humerus, radio, other	Stimulated	137 non-unions	74.5%

### Hip prostheses

Biophysical stimulation is an effective treatment for improving bone ingrowth in the presence of biomaterials and to prevent complications deriving from the failure of the implant, such as osteolysis.

In patients with painful uncemented hip prostheses, Rispoli et al. [46] reported a clinically evaluated success rate of good/excellent using PEMF treatment in 91% of those who used the treatment for more than 360 hours, compared to only 12% of non-compliant patients (< 360 hours). A few years later, Kennedy et al. [47] reported a 53% success rate in patients with femoral component loosening treated with PEMFs, compared to 11% of control patients. Dallari et al. [48] demonstrate that treatment with PEMFs eases the relief of pain and aids in clinical healing and the restoration of bone mass following revision total hip replacement.

### Vertebral fractures

**Fig. 6:**
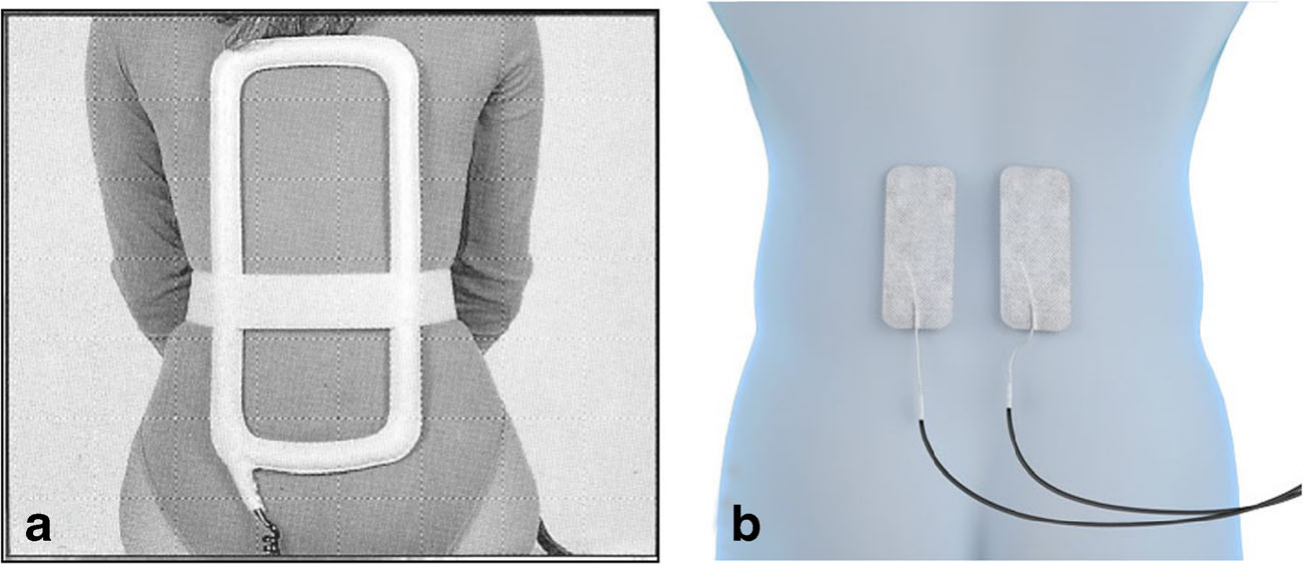
Representation of spinal stimulation with PEMF device (**a**) and CCEF device (**b**)

The first multi-centre study on 195 patients with anterior or posterior lumbar fusion reports a 92% success rate in the group stimulated with PEMFs, compared to 65% in the control group [49]. A few years later, Linovitz et al. [50] reported a 64% bone fusion rate in the active group after 9 months, compared to 43% in the placebo group (*p* < 0.003). Over the last few years, stimulation with CCEF has proved much more comfortable than inductive stimulation, due to the ease of use of the applicators, with fusion success rates of 84% [51] (Fig. 6). Beneficial effects on pain control and a reduced use of nonsteroidal anti-inflammatory drug (NSAIDs) are also described in literature. Rossini et al. [52] reported a significant pain reduction in the active group compared to placebo, with a consequent discontinuation of NSAIDs. Massari [53] and Piazzolla et al. [54] demonstrate an improvement in functional recovery following spinal fusion, and a significant reduction in the area of VBME in compression fractures respectively, with pain resolution times reduced by half.

### Osteonecrosis

Santori et al. [55] found the association of PEMFs, core decompression and grafts of trabecular bone tissue to be a valid solution in delaying or preventing collapse of the femoral head in the presence of osteonecrosis, with an 81% and 70% success rate in patients with Steinberg stage II and Steinberg stage III osteonecrosis, respectively. The most recent Italian study was conducted on 66 patients with osteonecrosis of the hip, stimulated with PEMFs for 8 h/day [56]. At two month follow-up, 53% of patients no longer reported any pain, and only 26% reported pain of moderate intensity. Radiography results showed a progression of the degeneration in only 26% of the hips studied (Table 4), and similar percentages have also been reported by Cebrian [57].

**Table 4:** Need for hip replacement by Ficat stage and progression of hip degeneration

Ficat	Hip replacement/number of hips	Number of hips (Ficat progression)
I	0/31 (0%)	3 (I ➝ II)
II	3/22 (14%)	5 (II ➝ III)
III	12/23 (52%)	12 (III ➝ IV)

### Summary

None of the authors of these studies suggest a blanket use of biophysical stimulation. In those cases in which the site, type of exposure, morphology of the fracture or condition of the patient indicate a risk of difficulties during the healing process, the use of biophysical stimulation is justified as a treatment to activate and finalise the process of osteogenesis and to speed the recovery process.

## Biophysical stimulation: clinical experience on joint

Pre-clinical research shows that treatment with PEMFs is anti-degenerative, helping to control local inflammatory phenomena and supporting cartilage repair processes in clinical setting (Table 5). Zorzi et al. [58] combined arthroscopic chondroabrasion and PEMF treatment. They showed that the percentage of patients assuming NSAIDs is significantly lower in those patients treated with PEMF compared to the placebo  group and functional scores were significantly better in the PEMF-treated group 90 days after surgery. The recovery times for functionality of the knee were significantly reduced in the short term and 87.5% of patients were unable to return to full sporting activity three years after the procedure in the placebo group, compared to 37.5% in the active group (*p* < 0.05). Benazzo et al. [59] have demonstrated that the percentage of patients using NSAIDs following ACL reconstruction is significantly lower in the patients treated with PEMFs compared to the placebo group and that the time required for recovery of knee function is significantly reduced in the short term. Two years after the reconstruction of ACL, a complete functional recovery was achieved by 86% of the patients in the active group compared with 75% of the patients in the placebo group. Similar results have been obtained following treatment using collagen scaffold seeded with bone marrow-derived cells for talar osteochondral lesions [60]. At six and 12 months follow-up, significantly higher AOFAS score and significant lower pain were recorded in the experimental group. More recently, Collarile et al. in patients following matrix-assisted autologous chondrocyte implantation (MACI) in the treatment of chondral lesions of the knee [61] show that patients in the PEMFs group had achieved a significantly better pain relief (2 and 6 months follow-up) and clinical outcome at the time of the 60-month follow-up. Two recent Italian studies [62, 63] on patients undergoing total knee arthroplasty have shown that PEMFs led to a significantly greater and more rapid reduction in post-operative pain symptoms as early as the first month and this was maintained at all follow-ups with a significant difference compared to the control group. The same observation was made for swelling. The study by Adravanti, which includes an evaluation 36 months after the procedure, shows that at this time-point only 7% of the patients in the PEMFs group still reported a level of persistent pain requiring the use of anti-inflammatory drugs, compared to 33% of control group patients. Furthermore, no patient in the PEMFs group expressed the need for walking aids compared to approximately 20% of the control group. A study conducted on patients in the early stages of osteoarthrosic degeneration of the knee, treated conservatively with PEMFs rather than surgically, showed significantly better results in terms of functional recovery and pain resolution at 12months follow-up [64]. The author concludes that an annual repetition of the treatment may result in sustained symptomatic improvement for the patient. Similar improvements in joint function, pain resolution and time needed to return to sporting activity were found in patients with patellofemoral pain following treatment with PEMFs [65]. The effect of PEMFs has also been studied in patients in the initial stages of spontaneous osteonecrosis of the knee [66], with results showing a significantly reduced level of pain after 6 months (*p* < 0.0001), an improvement in functional recovery and a rapid return to sporting activity. These results remained constant even after 24 months. The MRI evaluation at 6-month follow-up showed a significant reduction of total WORMS mean score (*p* < 0.0001).

**Table 5:** Summary of main clinical studies using pulsed electromagnetic field therapy on joint diseases

Author	Design of the study	Disease/treatment	Groups	Patients	Results	Long-term follow-up results
Marcheggiani Muccioli G [66], *European Journal of Radiology*. 2013	Case series	Spontaneous osteonecrosis of the knee	Stimulated	28	Pain relief, better functional recovery and necrosis area reduced	86% of knees preserved from prosthetic surgery at 2 years FU
Gobbi A [64], *Cartilage.* 2014	Case series	Early OA	Stimulated	22	Improvement in symptoms, knee function and activity	At 2-year follow-up, 80% of patients were satisfied and willing to repeat the treatment
Moretti B [62], *BMC Musculoskeletal Disorders*. 2012	Prospective comparative study	Grade 4 osteoarthrosis/total knee arthroplasty	Surgery + stimulated vs surgery	15 vs 15	Pain, joint swelling and knee score were significantly better and lower NSAID use	
Adravanti P [63], *International Orthopaedics*. 2014	Prospective comparative study	Grade 4 osteoarthrosis/total knee arthroplasty	Surgery + stimulated vs surgery	16 vs 17	Pain, knee swelling and functional score were significantly better	Severe pain and occasional walking limitations were reported in a lower number at 3 years FY (*p* < 0.05)
Cadossi M [60], *Foot & Ankle International*. 2014	Prospective comparative study	Osteochondral lesions in talar/bone marrow-derived cell transplantation	Surgery + stimulated vs surgery	15 vs 15	Pain relief, better functional recovery	
Iammarrone CS [65], *Bioelectromagnetics.* 2016	Prospective comparative study	Patellofemoral pain	Stimulated vs controlled	13 vs 17	Pain relief, better functional recovery and lower NSAID use	
Collarile M [61], *Knee Surg Sports Traumatol Arthrosc*. 2018	Prospective comparative study	Chondral knee lesions/matrix-assisted autologous chondrocyte implantation	Surgery + stimulated vs surgery	15 vs 15	Pain relief, better functional recovery	Better clinical outcome up to 5 years of FU (*p* < 0.05)
Zorzi C [58], *Knee Surg Sports Traumatol Arthrosc*. 2007	Randomised controlled double-blind study	Cartilage knee lesions, chondroabrasion/perforation	Active vs placebo	19 vs 12	Pain relief, better functional recovery and lower NSAID use	Completely recovered higher in the active group (*p* < 0.05) at 3 years of FU
Benazzo F [59], *Knee Surg Sports Traumatol Arthrosc*. 2008	Randomised controlled double-blind study	Anterior cruciate ligament lesion/reconstruction and meniscectomy	Active vs placebo	31 vs 29	Pain relief, better functional recovery and lower NSAID use	Complete functional recovery, no knee pain and return to sport activity higher in the active group (*p* = ns) 2 years of FU
Osti L, *International Orthopaedics*. 2015	Randomised controlled double-blind study	Grade III–IV cartilage knee lesions/partial medial meniscectomy and microfractures	Active vs placebo	34 vs 34	IKDC and Lysholm and constant scores were significantly improved in both groups with no significant intergroup differences	Clinical and functional outcomes were better in the PEMF-treated group at 5 years of FU
Osti L, *Orthopaedics.* 2017	Randomised controlled double-blind study	Small to medium rotator cuff tears/arthroscopic rotator cuff repair	Active vs placebo	32 vs 34	Pain relief, better ROM and stiffness and lower NSAID use	Clinical and functional outcomes were further improved in both groups, with no significant intergroup differences at 2 years of FU

### Summary

PEMF therapy can therefore be used proactively as (*i*) post-surgical treatment with the objective of quickly controlling local inflammatory response due to the surgical operation and, over the long term, to maintain the mechanical and biological properties of the cartilage or engineered tissue by means of an effective chondroprotective effect; (*ii*) post-arthroplasty treatment to inhibit the inflammatory processes that affect the periarticular tissues and to avoid the development of chronic pain and functional limitations; and (*iii*) conservative treatment to limit the progression of a degenerative process such as osteoarthritis that comes with age and is accelerated by inflammatory and/or traumatic events.

## Biophysical stimulation: future perspectives

Today, numerous other areas of medicine are preparing to use physical means to treat a variety of conditions or are seeing its potential. Some applications are in their infancy or are still at the stage of in vitro experimentation; however, current evidence seems to suggest that these treatment approaches will become increasingly widespread, for example in the treatment of tendinopathies or in neurology. The effects of PEMFs, in fact, have recently been studied on primary human cells isolated from semitendinosus and gracilis tendons exposed to PEMFs: results show that PEMFs do not alter cell vitality or induce apoptotic phenomena but are able to induce responses at gene expression level and to reduce the production of inflammatory cytokines in the tendon cells [67]. In MSCs isolated from the human umbilical cord and seeded in tendon differentiation medium, PEMF showed a greater production of collagen type I, scleraxis and greatest expression of tenogenic markers [68].

In neurology, there is great interest in the development of novel therapies for acute ischaemic stroke because thrombolysis is the only approved treatment. PEMFs could represent an alternative approach because of their effects on the main mechanisms of brain ischaemia. Capone et al. [69] demonstrated that PEMF can influence cortical excitability and do not produce side effects in healthy volunteers. Recently, a small, open-label, one-arm, exploratory study to evaluate the safety of PEMF stimulation in acute ischemic stroke (clinicaltrials.gov:NCT01941147) has been designed [70]. Preliminary results obtained in six patients demonstrated that a daily exposure of 120 minutes for five consecutive days is safe and tolerable.

## Conclusion

Biophysical stimulation is the result of solid scientific research. As reported in 2018 by Yuan et al., biophysical stimulation, as a prospective, non-invasive and safe physical therapy strategy to accelerate bone repair, has received tremendous attention in recent decades [71]. Moreover, the promotion effect has shown strikingly positive benefits in the treatment of various skeletal diseases. In the USA and Europe, research on the use of physical energy for bone repair processes has been ongoing throughout the past century. Every year, tens of thousands of patients undergo treatment all over the world. An inquiry to medical hospitals in the USA found that 72% of interviewed were offering biophysical stimulation to patients with fractures not yet healed at three months from trauma [72]. A substantial proportion of Canadian orthopaedic surgeons (45%) currently make use of bone stimulators as part of their management strategy for at least some tibial shaft fractures (for complicated tibial shaft fractures); 80% of respondents felt that a reduction in healing time of six weeks or more, attributed to a bone stimulator, would be clinically important [73]. More recently, in consideration of the sensitivity of cartilage tissue to physical stimuli, the orthopaedic community has now focused its interest on the joint to prevent cartilage degeneration, to enhance cartilage repair and to favour patients’ function recovery. Recently, Iwasa et al. have systematically reviewed the literature on the influence of PEMF in joints, including articular cartilage, tendons and ligaments, of publications from 2000 to 2016 [74]. The authors concluded that PEMF has a beneficial effect on chondrocyte proliferation, matrix synthesis and chondrogenic differentiation by upregulation of TGF-b and BMPs, and it decreases anti-inflammatory cytokines via A_2A_ and A_3_ adenosine receptors leading in clinical translational investigations, a beneficial effect on pain and functions of OA knees.

The orthopaedic community has undoubtedly played a central role in the development and understanding of the importance of physical stimuli to control biological activities. Orthopaedic research has demonstrated that the effects are dependent on physical parameters and, mediating from pharmacology, has introduced the concept of physical dynamics.

Compared to drug treatment, biophysical stimulation has the clear advantage that it can be administered locally with relative ease and reach the disease site at its maximum “concentration” and therapeutic efficacy, with no side effects. Biophysical treatment appears to be effective for the prolonged treatment of chronic degenerative conditions, while it does not seem suited to treating systemic diseases. An important aspect of treatment with physical agents is the ability to transfer the effects observed in basic research to clinical practice. Further development of the clinical use of physical agents involves overcoming numerous and complex hurdles; however, the ability to recognise and define an area with the potential for new treatment approaches, such as clinical biophysics, is a fundamental step in pointing the way for future research in various sectors.
